# Using Unsupervised Patterns to Extract Gene Regulation Relationships for Network Construction

**DOI:** 10.1371/journal.pone.0019633

**Published:** 2011-05-10

**Authors:** Yi-Tsung Tang, Shuo-Jang Li, Hung-Yu Kao, Shaw-Jenq Tsai, Hei-Chia Wang

**Affiliations:** 1 Department of Computer Science and Information Engineering, National Cheng Kung Tainan, Taiwan, Republic of China; 2 Department of Physiology, College of Medicine, National Cheng Kung University, Tainan, Taiwan, Republic of China; 3 Institute of Information Management, National Cheng Kung University, Tainan, Taiwan, Republic of China; University of New Orleans, United States of America

## Abstract

**Background:**

The gene expression is usually described in the literature as a transcription factor X that regulates the target gene Y. Previously, some studies discovered gene regulations by using information from the biomedical literature and most of them require effort of human annotators to build the training dataset. Moreover, the large amount of textual knowledge recorded in the biomedical literature grows very rapidly, and the creation of manual patterns from literatures becomes more difficult. There is an increasing need to automate the process of establishing patterns.

**Methodology/Principal Findings:**

In this article, we describe an unsupervised pattern generation method called AutoPat. It is a gene expression mining system that can generate unsupervised patterns automatically from a given set of seed patterns. The high scalability and low maintenance cost of the unsupervised patterns could help our system to extract gene expression from PubMed abstracts more precisely and effectively.

**Conclusions/Significance:**

Experiments on several regulators show reasonable precision and recall rates which validate AutoPat's practical applicability. The conducted regulation networks could also be built precisely and effectively. The system in this study is available at http://ikmbio.csie.ncku.edu.tw/AutoPat/.

## Introduction

Recently, a great amount of data about genes in various species have been produced and documented in the literature as a result of improvements in biological technology, like DNA microarrays and other high-throughput experiments for molecular biology. Thousands of genes can now be studied at one time. For biomedical scientists, it's an important issue to understand the relationship between aberrant gene expression and human diseases. To achieve this goal, transcriptional regulation of genes under normal and abnormal conditions needs to be established. The previous work that has received some attention in recent years is the protein-protein interaction finding tool from biological texts [1], [2], [3]. However, most bioinformatics tools are developed to analyze the relationship of gene-gene interaction and protein-protein interaction but not the specific transcription factor-target gene paradigm. We try to evaluate an interaction extraction tool, PIE [2], to find related sentences from annotated sentences of five transcription factors, i.e., E2F1, CREB, RAR-alpha, AP2 and ELK1. The average precision rate of extracted sentences is only 28.6%. These tools usually disregard the regulator and the target gene in gene regulation sentences. Due to this reason, it will result in enormously high false positive rates when applying these interaction finding tools to construct the gene regulatory network.

Also, more and more text mining studies in the biological domain do not only develop systems to discover gene-related findings in text, but also construct the specific interaction network because the need for network display and mining in the biological field is drastically increasing [4], [5], [6]. Therefore, it is necessary to develop a bioinformatics platform that focuses primarily on identifying transcription factor-target gene pairs so that a proper gene regulatory network can be established.

The biomedical literature documents a large scale of useful information and such biomedical knowledge is recorded in the plaintext format. These biomedical papers and literatures contain substantial gene-related information, including the transcriptional relationship between the transcription factor and its target genes. However, it takes lots of time for the researchers to acquire these relationships from the tremendous volume of sources. Moreover, the large amount of textual knowledge recorded in the biomedical literature grows rapidly, so the creation of manual patterns from literatures becomes a difficult and time-consuming task.

Some methods have been proposed to find gene-gene relationships from the biomedical literature. For example, the use of the gene co-occurrence method is popular [7], [8]. The base assumption is that genes which co-occur in the same literature frequently reflect an actual relationship between the two genes. Another approach uses document similarity. Each gene is linked to a kernel document and some documents that are most similar to any kernel document are then identified. This kind of document group is called the core document set. Two genes are linked if their core sets of the kernel documents have any intersection.

The associative concept space (ACS) has been developed for the representation of information extracted from biomedical literature [9], [10], [11]. The ACS is a multi-dimensional Euclidean space where thesaurus concepts are positioned, using co-occurrence of concepts as its source information. The distances between the concepts that are positioned in the space indicate their relatedness. To recognize the relationships in the literatures, the pattern matching method has also been proposed for extracting information on protein-protein interaction from scientific literatures [12]. The method does not use the complicated Natural Language Processing (NLP) technique. The author employs a protein dictionary, part-of-speech rules and word patterns to extract information on protein-protein interaction from scientific literature. These patterns are manually established. Gene Information System (GIS) is a biomedical text mining system that can retrieve gene-related information from PubMed [13]. In the second phase of GIS, called relation prediction, the authors predict the relationship between the gene pairs by the sentence expression pattern and the prediction rules that are generated with training samples. GIS determines the relation described in the sentences via the sentence expression patterns. Recently, PIE had been proposed for a protein-protein interaction (PPI) prediction system in text [2]. The PIE system utilizes natural language processing techniques and machine learning method to predict PPI sentences. It provides a Web service to extract PPIs from literature, including user-provided papers as well as PubMed articles.

Considering interaction extraction in the gene regulation mining issue, to the best of our knowledge, there are few studies which deal exclusively with gene regulation. Textpresso is the famous online gene-related mining system that included some categories with the regulation [14]. Furthermore, some groups have shown that there is a drastically increasing need to apply the text mining method to the gene regulation issue [15], [16]. These tasks have been focused on discovering specific rules manually for gene regulation mining from text. Saric et al. focused on detecting the noun phrase (NP) of biological entities that use the active and passive voices and proposed the NLP based method for regulatory relationship extraction [17], [18]. The previous studies on the interaction extraction task can be assigned to several categories. They established patterns or rules manually from literature or used NLP techniques to assist the patterns which are generated manually by domain experts from literature. Hahn et al. compared the rule-based system and the machine-learning-based system on the extraction of gene regulation events [19]. The compared results show that the rule-based system has better performance and the recall rate is highly affected by the machine-learning-based system. In this paper, we therefore aim to reduce the cost of manual rules for extracting gene expression relationships.

The method proposed in this article focuses on this aspect where the difficultly and the consumed time are reduced from the manual process to the automatic process. The goal of this work is to develop an unsupervised pattern generation module and use these patterns to extract gene expression relationships from literature for gene regulatory network construction. The system can establish unsupervised regulatory patterns automatically and retrieve the regulation relationship between the transcription factors and the target genes from literatures. The proposed gene expression relation mining system, AutoPat, use the statistical analysis and machine learning approach to construct the regulation patterns from un-annotated sentences that are extracted by a few annotated patterns. We then use the regulation patterns to construct the mining system. Given a particular query transcription factor, the system can extract the sentences that contain the regulation information of the query transcription factor from PubMed literature. We also show a regulation network construction framework based on the proposed system.

We preliminarily evaluated a baseline method to assess the difficulty of using unsupervised patterns. Three testing datasets were built from annotating transcription factors AP1, E2F1, and HIF-1 (the transcription factor hypoxia-inducible factor-1) related abstracts. These datasets contain 100, 107, and 241 positive sentences from 270, 279, and 619 sentences respectively.

We use 30 abstracts including the regulation relationship of HIF-1 to establish the verb set in advance. The verbs are keywords that may describe the regulation relationships according to the statistical information. These verbs were also defined as the action words [15]. Action words always describe relationships between the transcription factor and target gene. The baseline method “TF-KV-TG” is defined as judging the co-occurrence of a transcription factor, a key verb, and a target gene in a sentence. In the preliminary result, the baseline method can extract gene expression more precisely than other methods that extract the key entities or verbs only in sentences. However, the baseline method still suffers from a high false-positive rate. Therefore, in this paper, we aim to find a set of more precise patterns in an unsupervised manner to augment the baseline method.

We first use existing dictionaries to identify the transcription factor and target gene in the sentences. With the action word set, transcription factor, and gene names identification, we then manually establish several patterns that can describe the transcription factor and target gene regulation relationship from the answer sets to be our seed patterns. These seed patterns are then used to establish the unsupervised patterns automatically from PubMed.

In addition, we also take some linguistic features into consideration. These features and corresponding weights are obtained from the training data and are helpful in assisting the judgment of the correctness and importance of patterns. By exploiting the proposed unsupervised patterns and linguistic features, a weighted unsupervised pattern-based extraction system is then constructed. The system can effectively rank the sentences that have been matched by the unsupervised patterns.

## Materials and Methods

### Overall architecture of AutoPat

**Figure 1:**
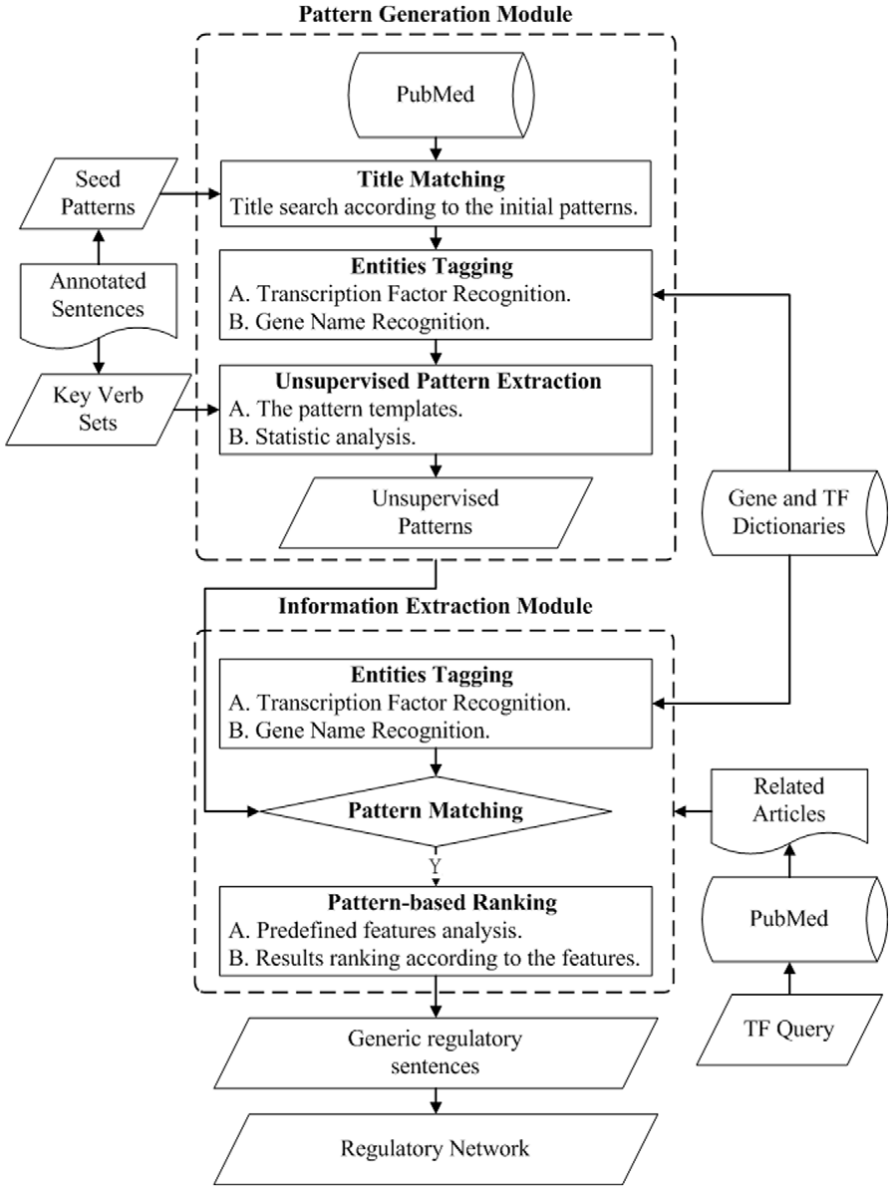
The overall architecture of AutoPat.

The proposed gene expression relation mining system, AutoPat, applied the weighted patterns to extract regulation relation information from literature. The overall architecture of AutoPat is shown in Figure 1. This system is composed of two major modules, i.e., the pattern generation module and the interaction extraction module. The pattern generation module uses a small set of supervised patterns to automatically search and build a large and comprehensive pattern set. After the unsupervised patterns have been established, these patterns are used for extracting the related regulation sentences from literatures in the interaction extraction modules. The system also provides a search interface to users for gene expression mining. Finally, the ranked sentences that contain the transcription factor and target gene expression are shown. AutoPat parses each sentence in the test dataset once and compares with a finite set of unsupervised patterns for extracting gene regulation related sentences. These processes take a linear time cost proportional to the number of input documents.

### Identification of transcription factors and gene names

To extract the regulation relationships from the literature, it is necessary to identify the transcription factor and gene names first. Here we used the existing dictionaries of the transcription factor and gene names to identify the transcription factor and gene names. The input term is defined as a *transcription factor* (TF) and all other terms that are found in the TF dictionary but not equal to the query are considered as *target genes* (TG). Therefore, for each query, the identified entities in a sentence contain one TF at most and the remaining entities are all considered as TGs. For recognizing TFs, the dictionary derived from Sequence Retrieval System (http://srs.ebi.ac.uk) is applied. All the factor names and their synonyms are taken into consideration. There are a total of 788 entries of TF data in the TF dictionary. An exception word set containing the ambiguous transcription factor names is also included. These terms are the same as common words in English, e.g. “*To*”, “*Alpha*” and “*Cell*”.

The gene dictionary we used for gene identification is derived from HUGO Gene Nomenclature Committee (http://www.gene.ucl.ac.uk/). The gene information we take into consideration includes the approved symbols, approved names, previous symbols and aliases. Some cases of approved names might contain the parentheses. For example, the approved name of ABCA4 is written as “*ATP-binding cassette sub-family A* (*ABC1*), member 4” in the HUGO database. We removed terms in the parenthesis and combine the remaining terms together. The original approved name is translated to “*ATP-binding cassette sub-family A, member 4*” and “*ABC1*”. This translation can reduce the amount of false-negative entities on the gene name recognition. The gene name dictionary contains total 22,995 entries of gene data.

### The pattern generation module

From the set of 30 abstracts of HIF-1 related articles, domain experts manually collected the key verb set and HIF-1 gene expression patterns. There are 60 sentences that describe the regulation relationships from these abstracts and total 88 key verbs and 80 seed patterns are manually extracted from these sentences. The seed patterns are all associated with the selected 88 key verbs and are used for the unsupervised pattern generation. In this paper, a gene expression pattern is assumed to contain at least one TG, one TF, and one key verb.

We then retrieved the abstracts with titles that contain these seed patterns from PubMed. We hypothesize that it is probable that an abstract will describe the regulation relationships if its title matches the seed patterns. The total number of retrieved abstracts is 10,761. These retrieved abstracts are then used to be our *training corpus* for the process of unsupervised pattern generation. In this process, three different kinds of *pattern templates* shown in Table 1 are established first. These templates are based on the arrangement of TF names, TG names, key verbs and prepositions where the prepositions are optional. By matching with the three templates, if a pattern in the training corpus that contains TF, TG, and the key verb terms also matches one of these templates, this pattern will be selected. It is noted that two or more pattern templates can be extracted in a sentence if the sentences of the retrieved abstracts contain multiple TFs, TGs, and key verbs. The set of selected patterns is called *unsupervised patterns* in this paper. For each template and extracted patterns, the numbers of occurrence in retrieved abstracts are counted. The set of patterns that describe the relation of the gene expression is then automatically constructed. In addition, a cut-off of pattern threshold is defined for our information extraction module. The assignment of the threshold will be discussed in result and discussion section.

**Table 1:** The pattern templates used for pattern generation.

ID	Pattern Templates
1	**[TF/TG] **+ **Key verb **+ (preposition) + **[TF/TG]**
2	**Key verb **+ (preposition) + **[TF/TG] **+ (preposition) + **[TF/TG]**
3	**[TF/TG] **+ (preposition) + **[TF/TG] **+ (preposition) + **Key verb**

From the selected abstracts, 3,514 unsupervised patterns are extracted. For the example pattern in PMID 9748288, a pattern “[TF/TG].*activation.*of [TF/TG]” will be conducted from the sentence “Recent reports described a role for the ***hyposia-inducible factor-1*** **(** ***HIF-1*** **)** in the transcriptional **activation of lactate dehydrogenase A**, *aldolase-A*, *phosphoglycerate kinase*, and *enolase-1 genes*.”. The symbol “*” means a wild card symbol that can match any word. In Table 2, the top 5 patterns of the pattern template 1 are shown according to their occurrence frequencies. We integrated the equivalent patterns to calculate their frequencies. For example, the pattern “TF/TG.*induced.*by.* TF/TG” is the subset of the pattern “TF/TG.*induced.* TF/TG”, so their frequencies are combined and the previous pattern will be removed from the set of unsupervised patterns. Because not all the patterns are useful, we used a frequency threshold to pick up proper patterns for which frequencies are beyond the threshold. In addition, four examples of seed patterns that include the same key verbs appeared in the top-5 unsupervised patterns of pattern template 1 are also shown in Table 2. This result shows that new patterns are generated by pattern generation module.

**Table 2:** Top 5 unsupervised patterns and examples of seed patterns of regulation relationships.

Top N	Unsupervised Patterns	#Occurrence
1	[TF/TG].*activation.*of.* [TF/TG]	1452
2	[TF/TG].*induction.*of.* [TF/TG]	1244
3	[TF/TG].*activate.* [TF/TG]	611
4	[TF/TG].*regulation.*of.* [TF/TG]	589
5	[TF/TG].*binding.* [TF/TG]	543

NO	Seed Patterns
1	[TF].*activator.*of.* [TG]
2	[TF].*induce.* [TG]
3	[TG].*regulated.*by.* [TF]
4	[TF].*binding.*site.*in.* [TG]

### The information extraction module

In previous studies, the statistics information between entities in a sentence is used as the key feature in extracting biological relationships, like protein-protein interaction [20]. In the pattern-based ranking strategy, several pattern related features are evaluated to rank the final result. We gathered the statistical results of distribution of the distances, i.e. number of words between TFs and TGs among the positive and negative sentences. There are 60 sentences which are used for seed pattern generation are also used for the preliminary evaluation of unsupervised patterns. From these 60 sentences, we compare the numbers of sentences that matched with seed patterns and unsupervised patterns. There are 45 positive sentences that match the seed patterns and 57 positive sentences that match the unsupervised patterns. The distances in most positive sentences are less than 10. Only a few cases in the positive sentences have a distance of more than 10. The genes in the sentences matching the patterns may not be the target genes of the TFs in the sentences if the TFs are too far away from the genes in the sentences. Because many sentences have very complicated structures and the regulation relationships described in some sentences are ambiguous, it is difficult to extract the regulation relationships from the sentences using only pattern matching. Therefore, we also take other features into consideration. In addition to pattern matching score, we also integrated the effects of TF-TG distance, the position feature, type of pattern template, and number of TF, TG and Key verb into our ranking strategy.

We gathered statistical information about the positions of the correct sentences in the abstracts from the training set. From the experimental statistical results, it is obvious that the sentences in the titles or in the final part of the abstracts have higher probabilities of describing the regulation relationships between TFs and TGs. The number of sentences that are in the preceding part of the abstracts is much fewer than others. This means that fewer TF-TG regulation relationships are mentioned in the earliest part of the abstracts. Therefore, we assign the sentence a position weight if the sentence is in the title or in the final part of the abstract.

We used features of pattern matching, position of the sentence in the abstract, the distance between TF and TG, number of TF, TG, and Key verb, and type of pattern template in the ranking strategy. In feature weight assignment, the pattern that matches the pattern template 1 has a higher probability of describing regulation relationships than those matching the pattern template 2 and 3, because the patterns of template 1 are more meaningful than those of type 2 and 3. Therefore, only template 1 attains the pattern match weight.

**Figure 2:**
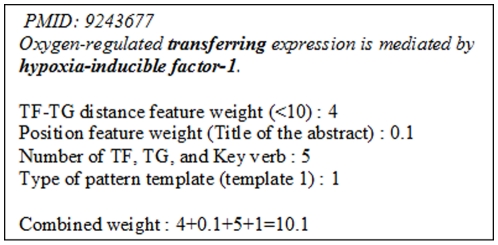
Example of combined weight of extracted sentences.

Each feature weight is assigned according to the statistic information of gene regulation sentences. After each feature is assigned a proper weight according to the meaning it represents, we can integrate the information of the sentences and calculate the combined weight of each sentence. The combined weight is defined as the sum of each feature weight. According to the statistics information of each feature, we hypothesized that a sentence consists of these four features will has a higher likelihood of describing the regulation relationships. Figure 2 shows an example of counting the combined weight for an extracted sentence. Each assigned weights are predefined for combined weight. In this example, the TF-TG distance is less than 10 and the assigned weight is 4. Overall, the combined weight is 10.1.

This weight can be used to representing the probabilities of the sentences in describing the regulation relationships. Because not all the extracted results are promised to be correct, the final results will be sorted by the ranking method.

## Results and Discussion

### Unsupervised pattern validation

Two testing sets are used to evaluate the extracted unsupervised patterns. The first one (A) is the original training set, i.e., 60 sentences which are used to build the seed patterns. The second one (B) is also related to “HIF-1” and contains 26 sentences. These 26 HIF-1 related sentences are collected from PubMed keyword search and annotated by domain experts. The precision rates of unsupervised patterns in Dataset A and Dataset B are 95% and 92% respectively while recall rates are 100% and 96%. The experiment results show that a high precision rate for the unsupervised patterns is achieved and more correct sentences can be found. The results verify that our proposed method is able to extract extra useful patterns from a large amount of unsupervised data.

**Figure 3:**
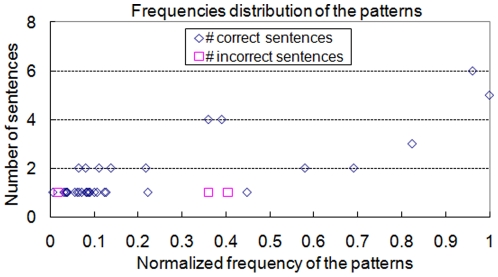
The frequency distribution of unsupervised patterns.

**Figure 4:**
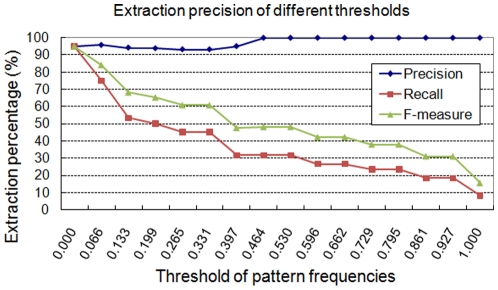
The extraction precision of different thresholds.

In addition, we use the extracted results to verify the relationship between correct sentences and highly frequent unsupervised patterns and to determine the threshold of unsupervised patterns for the following experiments. The frequency distribution of the unsupervised patterns is therefore also evaluated. The result is shown in Figure 3. The frequencies of the patterns are normalized by dividing by the maximal frequency, 1,510. This result shows that even though the incorrect sentences match the patterns, the patterns they match have lower frequencies. In addition, the correctness of extraction result is very important to biologists because thousands of genes may have associations with each other but not specific gene regulation relationships. Therefore, we consider not only the higher F score but also the higher extraction precision. We calculated the precision rates of the unsupervised patterns under different thresholds of the frequencies and the result is illustrated in Figure 4. When the threshold is raised to the value 700, which has a normalized value of 0.464, the precision rate can be increased to 100% while lots of False Positive (FP) cases are then filtered from extraction result. Moreover, the goal of our system is to construct a gene regulation network from literature. We also observed that many TGs in result sentences that are extracted by low-frequency patterns can also be found in sentences extracted by high-frequency patterns. Therefore, due to the merit of the high precision rate and the reduction of computation cost, the threshold of unsupervised patterns is set to value 700 for the following experiments.

**Figure 5:**
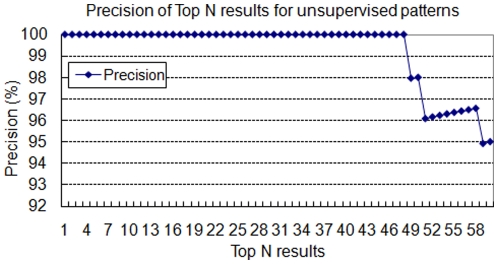
The precision of the Top N ranking results for unsupervised patterns.

However, some extraction errors still arise. Certain correct sentences that contain the regulation relationship are missed by our method due to the lack of the key verbs. Such kind of sentences often contains general verbs, such as “identify” and “demonstrate”, or describes the regulation relationship by using a clause. A sentence “Sequence analyses identified **Hif-1-binding** sites in the promoters of ***MCP-1*** and ***MCP-5*** genes.” From PMID 17474992 is used as an example for illustration. This sentence describes the regulation relationship without using any key verbs. Because we assumed that the correct sentences should contain at least one key verb, the sentences without key verbs will not be extracted by the system. The precision rate of the ranking results is shown in Figure 5. The proposed ranking method is useful to distinguish the correct sentences from the incorrect sentences.

**Figure 6:**
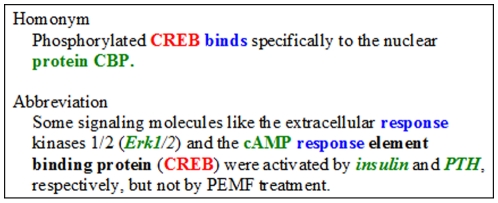
Examples of the homonym and abbreviation issues.

We also use five more TF queries, i.e., E2F1, CREB, RARalpha, AP2, and ELK1 to test our system and the key verbs are manually collected from 30 abstracts that are also used in generating 80 seed patterns. However, when the larger testing data is applied to AutoPat, two name entity recognition problems, i.e., *homonym* and *abbreviation*, have been observed in the extraction process. In the homonym case, a tagged name could be found in both the gene dictionary and TF dictionary. The TF name has been tagged as a gene name according to dictionaries in many sentences. The ratio is about 20% in the homonym case. Furthermore, the TF has the most important role in the regulatory process. In our tagging parameter, the TF has higher priority than gene. In the abbreviation case, a gene or a protein name usually has a full-name form. The abbreviation issue will happen while some key verbs or other abbreviation words appear in the full-name form. The ratio is about 5%. Examples of homonym and abbreviation cases are shown in Figure 6. The protein CBP is not a gene name in the first sentence. The term “response” appeared in the full form of CREB is not a correct key verb in the second sentence. These sentences are all incorrect tagging results. Homonym and abbreviation adjustments are proposed for improving the accuracy in tagging TF and TG names. The overall average improvement of the precision rate is close to 20%. The adjusted system is used as the mature version in the following experiments.

Next, the mature version of AutoPat was compared with the famous relation extraction system in our preliminary evaluation, Textpresso. In Textpresso, users can specify the different biology category for relation extraction. We selected categories in Textpresso that have a similar biological relation to gene expression, e.g., “regulation”, “spatial relation” and “action” categories. Besides, we also compare the performance of the joined categories in Textpresso. In this comparison, the extracted sentences of gene expression relationship of Textpresso and AutoPat were both evaluated by domain experts. The precision and recall rates of Textpresso and AutoPat in HIF-1 TF are shown in Table 3. The recall and precision rates of Textpresso are low for all test categories. The precision rate decreased by joining these three categories, and the recall also not highly increased. Overall, our method achieved a high precision rate and a better recall than Textpresso.

**Table 3:** The performance comparison with Textpresso.

	Precision	Recall	F-measure
Textpresso-Regulation	42.5%	22.9%	29.7%
Textpresso-Spatial	45.5%	6.2%	10.9%
Textpresso-Action	32.0%	9.2%	14.3%
Textpresso-Join	27.7%	26.4%	27.0%
AutoPat	**95.0%**	**65.5%**	**77.5%**

The top-k precision rate is then used to assess the effectiveness of the unsupervised patterns and the pattern-based ranking strategy. Results in Table 4 have shown that the new generated pattern and the ranking strategy can improve the precision rate of the top answers.

**Table 4:** Top-K Precision of AutoPat.

Top-K	AP1	E2F1	Average
10	100%	70%	85.0%
20	85%	85%	85.0%
30	70%	70%	70.0%
40	70%	57%	63.5%
50	68%	58%	63.0%
R-Precision	57.3%	60.5%	58.9%

Note that the precision rates of baseline “TF-KV-TG” for AP1 and E2F1 are 48.1 and 53.3.

### Performance evaluation

One of extraction methods in previous studies, Saric's rule-based method is used as our comparison target for evaluating AutoPat's performance. Recently, many studies used the NLP parser to construct the dependency tree for extracting biological relationships. Their main idea is to extract the sub-paths between two entities from the dependency tree. A path should contain at least one biological action word (Key Verb). In our experiments, we simulated Saric's rule-based extraction method by using Stanford parser [21], [22]. In addition to the original seed pattern collected by HIF1 TF, we also collected another set of mixed manual annotated training sentences of TFs, i.e., AP2, CREB, E2F1, ELK1, and RAR-alpha, to be one set of manual training sentences for the construction of seed patterns. In total, there are 76 seed patterns from 80 annotated mixed sentences, and this set is represented as “MIX” in Table 5. A filter TF was used to select the related abstract from the training corpus. Results from the different filtering selection could validate the applicability of established unsupervised patterns. The results of Saric's method show a lot of correct relationships that contain nouns of key verbs are filtered out by NP detection. In AutoPat, a lot of sentence patterns were trained by the pattern generation module. The preposition and orders between entities and verbs, i.e., the noun forms of key verbs, were learned in our sentence patterns. The result shows that AutoPat does not have the large recall gap with trained NP sentence patterns. In other words, AutoPat does not lose a lot of correct relationships of NP in biological sentences. Overall, our recall is not only better than Saric's method but also has a smaller gap of precision.

**Table 5:** The overall performance comparison.

Testing Data	Method			Precision	Recall	F-measure
AP1 (270)	Saric's method			54.3%	25.0%	34.2%
	AutoPat	Seed	Filter			
		MIX	None	49.2%	70.8%	58.1%
			AP1	46.0%	64.4%	53.6%
			E2F1	49.2%	64.4%	55.8%
			HIF1	48.0%	69.1%	56.7%
		HIF1	None	45.7%	60.7%	52.2%
			AP1	48.3%	64.0%	55.1%
			E2F1	52.1%	69.7%	59.6%
			HIF1	47.8%	85.4%	61.3%
E2F1 (279)	Saric's method			58.5%	57.9%	58.2%
		Seed	Filter			
	AutoPat	MIX	None	60.5%	70.5%	65.1%
			AP1	61.7%	72.4%	66.6%
			E2F1	62.6%	66.3%	64.4%
			HIF1	58.9%	70.6%	64.3%
		HIF1	None	57.9%	62.9%	60.3%
			AP1	58.5%	59.6%	59.0%
			E2F1	56.4%	54.8%	55.6%
			HIF1	53.7%	84.6%	65.7%
H1F1 (619)	Saric's method			50.8%	24.4%	33.0%
		Seed	Filter			
	AutoPat	MIX	None	55.3%	45.6%	49.9%
			AP1	52.8%	45.0%	48.6%
			E2F1	54.3%	42.2%	47.5%
			HIF1	50.6%	49.0%	49.7%
		HIF1	None	52.7%	42.6%	47.1%
			AP1	56.3%	38.5%	45.7%
			E2F1	59.6%	38.5%	46.8%
			HIF1	52.7%	57.7%	55.1%

The TFs in the “Filter” column are used to select the related abstracts from the training corpus.

We also used the Learning Language in Logic (LLL) dataset [23], [24]. The LLL dataset is a publicly available dataset that contains gene interaction annotations and has been used frequently in recent work on biological relation extraction. For LLL dataset, the F-measures of Saric's method and AutoPat are 39% and 60% respectively.

For the evaluation of gene expression network construction, we evaluated the constructed network with pathways in the Pathway Interaction Database (PID). PID is the integrated online database that contains multiple curated interaction pathways composed of human molecular signaling and regulatory events and key cellular processes [25]. PID was created by a collaboration between the US National Cancer Institute and Nature Publishing Group and serves as a research tool for those interested in cellular pathways. We used the related abstracts from literature of HIF-1, E2F1 and AP1 transcription factor pathways that were collected from PID to evaluate AutoPat. The number of known TG nodes of HIF-1, E2F1 and AP1 transcription factor pathways in PID are 45, 30 and 47, respectively. The extracted results and performance evaluation of AutoPat and Saric's method are listed in Table 6. The numbers of known TGs of HIF1, E2F1 and AP1 extracted by AutoPat are 29, 18 and 32, respectively. Because some abstracts do not have enough information for our method to extract the correct answer, several TG nodes could not be extracted. For examples, some known TGs in PID are not mentioned in abstracts and no aliases or synonyms of HIF-1 are described directly in the abstract of CAIX TG. In sum, there are twelve TGs that AutoPat cannot correctly extract from abstracts. This is because their regulation relationships are usually described in multiple sentences. Information in a single sentence is not enough to judge the relation.

**Table 6:** The list of extraction results of HIF-1 TF pathway in PID.

TF		Method	Target Gene
HIF-1	Found	AutoPat (**29**)**P: 64.2% R: 80.5%**	*ET1, * ***MDR1*** *, beta_integrin, CD73, TF, * ***PFKFB3*** *, Leptin, * ***TERT*** *, MCL1, TFF3, CP, FURIN, DEC1, ALDOA, ENO1, ID2, * ***ABCG2*** *, * ***PFKL*** *, * ***CITED2*** *, * ***FECH*** *, ETS1, DEC2, * ***BNIP3*** *, TfR, * ***CXCL12*** *, * ***HMOX1*** *, * ***RORA4*** *, * ***NOS2*** *, EPO*
		Saric's method (**19**)**P: 48.6% R: 52.8%**	*ET1, beta_integrin, CD73, TF, Leptin, MCL1, CP, FURIN, DEC1, ALDOA, ENO1, ID2, ETS1, DEC2, TfR, RORA4, EPO, * ***PHD2*** *, * ***GLUT3***
	Not Found	not in abstract (**9**)	*PGK1, ADRP, NDRG1, PGM1, PKM, ADM, HK2, HK1, CAIX*
		in abstract, AutoPat (**7**)	*PHD3, NPM1, PHD2, IGFBP1, GLUT3, PAI, CXCR4*
		in abstract, Saric's method (1**7**)	*PHD3, NPM1, IGFBP1, PAI, CXCR4, MDR1, PFKFB3, TERT, TFF3, ABCG2, PFKL, CITED2, FECH, BNIP3, CXCL12, HMOX1, NOS2*
E2F1	Found	AutoPat (**18**)**P: 63.3% R: 78.3%**	***XRCC1*** *, HIC1, * ***MCL1*** *, SIRT1, * ***APAF-1*** *, SP1, DHFR, KAP1, * ***E2F2*** *, CDC25A, E2F1, P21CIP1, * ***RB1*** *, PAI, Cyclin-D3, * ***uPA*** *, P73, CDK1*
		Saric's method (**14**)**P: 36.5% R: 60.9%**	*HIC1, SIRT1, SP1, DHFR, KAP1, CDC25A, E2F1, P21CIP1, PAI, Cyclin-D3, P73, CDK1, * ***MAD2*** *, * ***p14ARF***
	Not Found	not in abstract (**7**)	*HST, carboxylesterase, Caspase-7, TK1, Cyclin-E, MCM3, HSORC1*
		in abstract, AutoPat (**5**)	*MAD2, WASF1, p107, Cyclin-A, p14ARF*
		in abstract, Saric's method (**9**)	*WASF1, p107, Cyclin-A, XRCC1, MCL1, APAF-1, E2F2, RB1, uPA*
AP1	Found	AutoPat (**32**)**P: 67.9% R: 71.1%**	*EGR1, TH, * ***Fra2*** *, * ***GR*** *, IL2, CYR61, IL8, * ***Connexin43*** *, FOS, * ***p53*** *, * ***MMP1*** *, * ***TIMP1*** *, ETS1, IL5, Angiotensin II, MYC, * ***ANF*** *, proenkephalin, PTEN, * ***MMP9*** *, * ***p27Kip1*** *, IL10, * ***GM-CSF*** *, * ***MKP1*** *, * ***A-FABP*** *, CDK1, * ***Cyclin D1*** *, * ***ER-alpha*** *, ET1, IL4, * ***uPA*** *, Dmp1*
		Saric's method (**22**)**P: 35.2% R: 48.0%**	*EGR1, TH, IL2, CYR61, IL8, FOS, ETS1, IL5, Angiotensin II, MYC, proenkephalin, PTEN, IL10, CDK1, ET1, IL4, Dmp1, * ***CCL2*** *, * ***COL1A2*** *, * ***IFN-gamma*** *, * ***Myb*** *, * ***TCF4***
	Not Found	not in abstract (**2**)	*DMTF1, BIM*
		in abstract, AutoPat (**13**)	*Neurotesin, MHC-1A, CCL2, TGFB1, Actin, TCF4, Myb, Fra1, PEBPB2, p16INK4a, COL1A2, IFN-gamma, MT2A*
		in abstract, Saric's method (**23**)	*A-FABP, Actin, ANF, Connexin43, Cyclin D1, ER-alpha, Fra1, Fra2, GM-CSF, GR, MHC-1A, MKP1, MMP1, MMP9, MT2A, Neurotensin, P16INK4a, p27Kip1, p53, PEBPB2, TGFB1, TIMP1, uPA*

*P: Precision, R: Recall, the bold-faced target gene means this TG can be extracted in only one method.*

**Figure 7:**
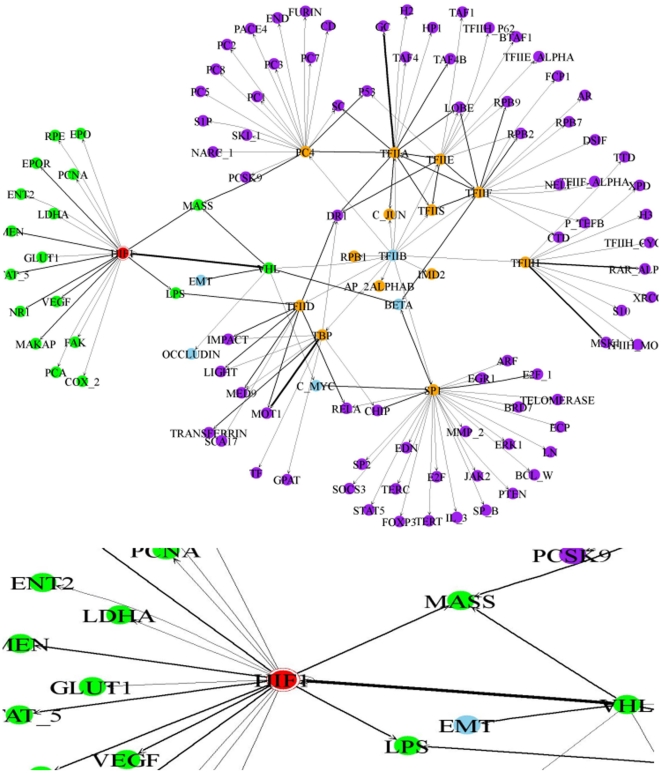
The global network of HIF-1 TF.

**Figure 8:**
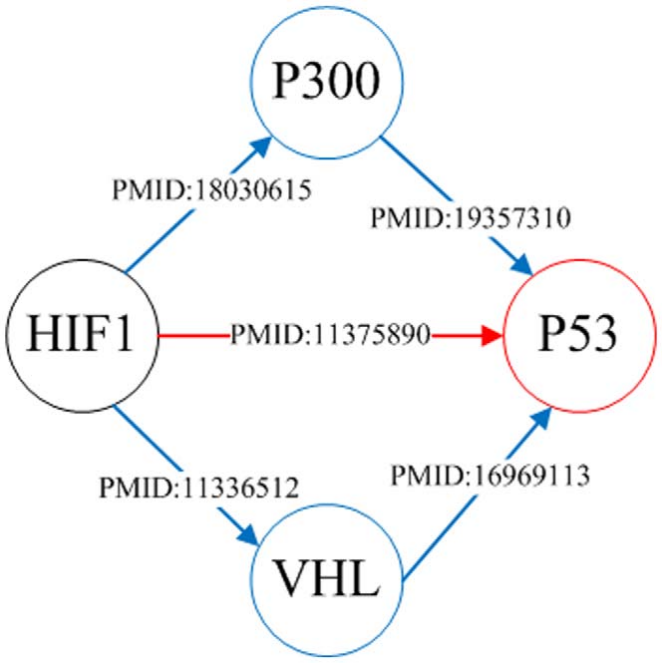
The example of an indirect relationship between HIF-1 and *p53*.

In Figure 7, a four layers global gene regulatory network that contains direct and indirect relationships with HIF-1 TF and its partial network are shown. After the first TG set of HIF-1 is extracted, their confidence values are conducted by the occurrence frequency. If an extracted TG is also found in the TF dictionary, this TG is considered as the next TF for extracting generic regulatory relationships. The process is repeated four times. The extracted relationships are shown as a directed graph. The nodes represent TFs or TGs and the arcs are represented pairwise regulation relationships. An arc points from TF to TG and the type of relation is indicated as the generic regulatory relation. TGs in the layer one are denoted by green nodes. Furthermore, the blue, orange, and purple nodes indicate the other TGs from layer two to layer four. A high confidence regulation relation is denoted by a bold line. In this network, five well-studied regulation relationships are found by high confidence arcs. The *VHL* TG is regulated by HIF-1 TF directly in the layer one. The *GC*, *RAR-alpha*, *MSK-1*, and *MOT1* TGs are indirectly related to HIF-1 TF in the layer four through some important nodes. *VHL* and *TFIIB* are found as important nodes between these indirectly related TGs with HIF-1 TF. The overall pathway information is listed in Table 7. Figure 8 shows the instances of direct and indirect generic regulatory relationships from HIF-1 TF to *P53* TG. HIF-1 regulates *P53* gene directly according to the articles (PMID: 11375890). Besides, *P53* gene is also regulated by HIF-1 indirectly through *P300* or *VHL* regulation processes.

**Table 7:** The high frequency of TF-TG relationships in HIF-1 global network.

Query TF	Middle Nodes	TGs	Relation Type
HIF-1		*VHL*	Direct
	*VHL (layer 1)*	*GC*	Indirect
	*TFIIB (Layer 2)*		
	*TFIIA (Layer 3)*		
	*VHL (layer 1)*	*RAR-alpha*	Indirect
	*TFIIB (Layer 2)*		
	*TFIIH (Layer 3)*		
	*VHL (layer 1)*	*MSK-1*	Indirect
	*TFIIB (Layer 2)*		
	*TFIIH (Layer 3)*		
	*VHL (layer 1)*	*MOT-1*	Indirect
	*TFIIB (Layer 2)*		
	*TBP (Layer 3)*		

### Conclusions

In this paper, we designed and developed an unsupervised pattern generation method and an information retrieval system, AutoPat. This system is able to establish patterns automatically and retrieve the regulation relationships between the transcription factor (TF) and target genes (TG) from the PubMed literature using unsupervised patterns for gene expression network construction. Although AutoPat cannot distinguish whether the second TF is a TF or a TG, this is still a correct sentence because in Biology, self regulation does exist. The concept of our proposed method can also be applied to other relationship extractions between biological entities such as protein-protein interaction. The extracted results are sorted according to the score assigned to each sentence, in order to save time for users to view the extracted sentences. Because the sentence patterns used to describe regulation relationships in the literatures are about the same for each TF, therefore, our proposed method can also achieve high accuracies for other TFs. Experiments on several TFs show reasonable precision and recall rates which validates AutoPat's practical applicability. In the future, the incremental pattern mining topic will be considered for biomedical literature mining.
